# Bacterial Niche-Specific Genome Expansion Is Coupled with Highly Frequent Gene Disruptions in Deep-Sea Sediments

**DOI:** 10.1371/journal.pone.0029149

**Published:** 2011-12-21

**Authors:** Yong Wang, Jiang Ke Yang, On On Lee, Tie Gang Li, Abdulaziz Al-Suwailem, Antoine Danchin, Pei-Yuan Qian

**Affiliations:** 1 KAUST Global Collaborative Research, Division of Life Sciences, Hong Kong, University of Science and Technology, Clear Water Bay, Hong Kong, China; 2 Institute of Oceanography, Chinese Academy of Sciences, Qingdao, China; 3 King Abdullah University of Science and Technology, Thuwal, The Kingdom of Saudi Arabia; 4 AMAbiotics, SAS and CEA/Genoscope, Evry, France; Brigham Young University, United States of America

## Abstract

The complexity and dynamics of microbial metagenomes may be evaluated by genome size, gene duplication and the disruption rate between lineages. In this study, we pyrosequenced the metagenomes of microbes obtained from the brine and sediment of a deep-sea brine pool in the Red Sea to explore the possible genomic adaptations of the microbes in response to environmental changes. The microbes from the brine and sediments (both surface and deep layers) of the Atlantis II Deep brine pool had similar communities whereas the effective genome size varied from 7.4 Mb in the brine to more than 9 Mb in the sediment. This genome expansion in the sediment samples was due to gene duplication as evidenced by enrichment of the homologs. The duplicated genes were highly disrupted, on average by 47.6% and 70% for the surface and deep layers of the Atlantis II Deep sediment samples, respectively. The disruptive effects appeared to be mainly due to point mutations and frameshifts. In contrast, the homologs from the Atlantis II Deep brine sample were highly conserved and they maintained relatively small copy numbers. Likely, the adaptation of the microbes in the sediments was coupled with pseudogenizations and possibly functional diversifications of the paralogs in the expanded genomes. The maintenance of the pseudogenes in the large genomes is discussed.

## Introduction

Bacteria employ different strategies to achieve fitness and to adapt to different environments, as shown in the deciphered genomes of microbes from rare niches [1], [2]. Bacteria living in complex environments like soil are often equipped with complex genomes characterized by frequent occurrences of recombination, rearrangement, gene amplification, viral insertion and horizontal gene transfer [1], [2]. These large genomes with sizes greater than 9 Mb have been deemed to be a result of the selection pressure created by the scarcity and diversity of nutrient sources and low penalties for slow growth in the soil [3]. The processes that lead to genome expansion mainly include integration of foreign genes and amplification of gene families [4], [5]. In contrast, small genomes (size<1 Mb) were generally found in symbiotic and chemolithotrophic bacteria [2]. The reductive selective forces lead to gene loss, gene transfer to the host genome and short intergenic spaces for efficient metabolic streamlining [4]. In fact, reports have shown that bacteria from the same phylum have a wide range of genome sizes because of their adaptation to different niches [4], [6]. In brief, bacterial genomes can be strongly shaped by their environments.

Bacteria in complex environments adopt mutations, expression regulation and gene duplication to cope with stresses to various degrees. Gene duplication is a frequent means to cope with steep gradients of variable toxic factors (reviewed in [7]). If relevant forces exist, the functions and copy number of the duplicated genes may persist and some may develop new functions [8] as originally proposed by Ohno [9]. Pioneering research has demonstrated cases of gene duplication and subsequent steps to gain selective advantages among bacterial strains [7]. It has been suggested that gene duplication is more efficient than point mutation in improving fitness of a microbe that confronts a sudden change in certain factors such as temperature and salinity [7]. Gene duplication benefits the microbe by enhancing the number of protein factors that are vital to coping with environmental challenges over a short period of time. As a result of gene duplication, expansion of microbial genomes has been observed in some extreme or complex environments such as hyperthermal water and soil [10], [11].

Under stable conditions, drastic genome modifications rarely occur, particularly because genomes found in such situations carry a variety of antimutator genes [12]. Mutations generally interpreted as neutral represented the majority of genomic changes in a laboratory population of *Escherichia coli* across 20,000 generations [13]. In contrast, gene expansion due to gene duplication has been systematically revealed among natural bacterial and archaeal populations isolated from different niches [14]. In short, bacterial genomes are dynamic in complex environments experiencing steep physico-chemical gradients, as the evolutionary processes have to be rapid and extensive when bacteria migrate across the interface of two different environmental settings. However, the corresponding dynamics and processes have not been described in detail, because 1) the genomes of the relevant communities have not been sequenced, 2) many of the bacteria are not culturable in any straightforward way, and 3) fast-evolving bacteria from the same lineages have not been explored in terms of genomic adaptation and gene duplication. Among culturable bacteria isolated from extreme environments, the genomic features related to environmental stresses might be rapidly lost after several generations of growth in the laboratory, as a consequence of alleviating the previous selection pressure. An example of this process is known in the case of the resistance of *E. coli* to ampicillin [15]. Thus, the bacteria cultured in laboratories for many generations may not be best suited for studies of their original genetic features.

Metagenomic studies aiming at identification of in situ microbial communities in niches displaying steep physico-chemical gradients have improved considerably with the steady improvement of computation capacity and pyrosequencing techniques (reviewed in [16]). A large number of approximately 400 bp genomic sequences (reads) can be obtained for a microbial community sampled from an environment of interest in a 10-hour run on a 454 platform [17]. Microbial genomes from a variety of environmental settings have been investigated in terms of genomic size variations, gene duplication, and the spread of transposases [10], [18]. Thus, the genomic features of the microbial inhabitants of an unexplored site can be connected to environmental factors [19]. Moreover, the evolutionary processes revealed by metagenomes belonging to the same community evolving in response to changes in physico-chemical factor can be examined.

**Figure 1:**
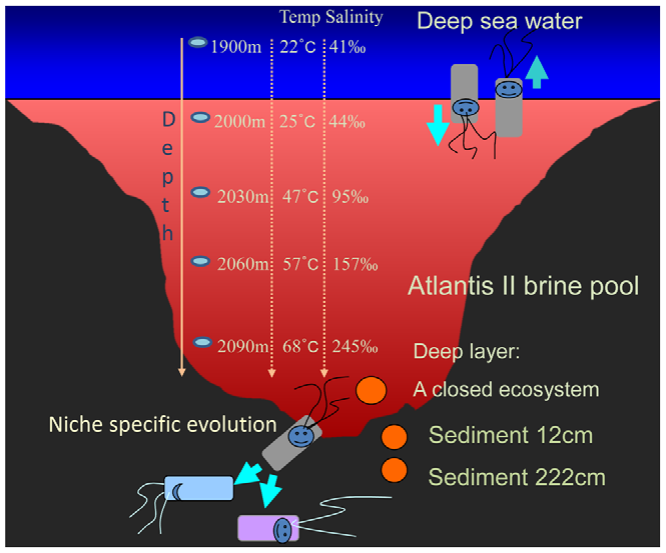
Schematic of the Atlantis II Deep brine pool and hypothetical bacterial niche-specific evolution. The figure briefly describes the vertical changes of the temperature and salinity in the Atlantis II Deep brine pool. Brine water and a sediment core were collected from the deep layer of the brine pool and the seafloor, respectively. Microbial samples were then obtained at depths of 12 cm and 222 cm from the core. Free microbe exchanges occur at the interface of the seawater and brine pool, all under natural selection; the microbial lineages in the deep layer (a closed ecosystem) heavily evolve during their adaptation to the sediment.

The deep-sea hydrothermal systems in the Red Sea are unique and extreme environments. The microbes in these extreme habitats have not yet been fully characterized [20], [21]. Since its discovery in 1965, the Atlantis II Deep brine pool has been described as an anaerobic, hypersaline, and metalliferous environment. The temperature of the brine pool has increased from 56°C to 71°C in recent decades [22], [23], [24]. From the brine-sea water interface to the bottom of the brine pool, there are three upper convective layers and a lower layer characterized by strong temperature and chemical and metal ion gradients. An internal brine circulation is driven by hot influx at the bottom of the pool [21]. The Atlantis II Deep sediment is a relatively closed ecosystem because the exchange of species and matters with the overlying seawater was rather limited (Fig. 1). The bacteria in the deep layer are almost all chemoautotrophic, using aromatic compounds and metals as energy and nutrient sources [25].

The microbes colonizing the sediment originate from those inhabiting the deep layer of the brine pool. The sediments in the Atlantis II Deep brine pool are soft muds filled with halite, anhydrate, metal oxides and up to 97% pore water [26], which permits deep dispersal of the microbes from the brine pool into the sediments. In parallel, the genomes of the microbes evolve accordingly in a relatively stable niche (the sediment) (Fig. 1). As such, we hypothesized here that the genomic repertoire in the sediments would reflect adaptation to the sediment environment. In this study, we pyrosequenced the metagenomes in the water and sediments in the Atlantis II Deep brine pool and identified the open-reading frames (ORFs) in the pyrosequencing reads for the genes listed in the Kyoto Encyclopedia of Genes and Genomes (KEGG) database [27]. Estimation of effective genome sizes indicated that the microbes in the Atlantis II sediment samples had undergone genome expansion through gene duplication. To demonstrate completeness and variants of the duplicated genes, a simulation-based method was developed to estimate their disruption rates. The model showed that a large fraction of the abundant genes from the surface and deep layers of the Atlantis II Deep sediment were pseudogenes and functional variants. This modeling result was supported by additional surveys of the ORFs based on Blast results. The gene duplications were highly frequent across variant gene families in the microbial lineages in the Atlantis II Deep pool. These results suggest that a bacterial genome can be rather dynamic and complex in response to tightening and relaxing of environmental stresses.

## Results

### Microbial communities and effective genome size

**Figure 2:**
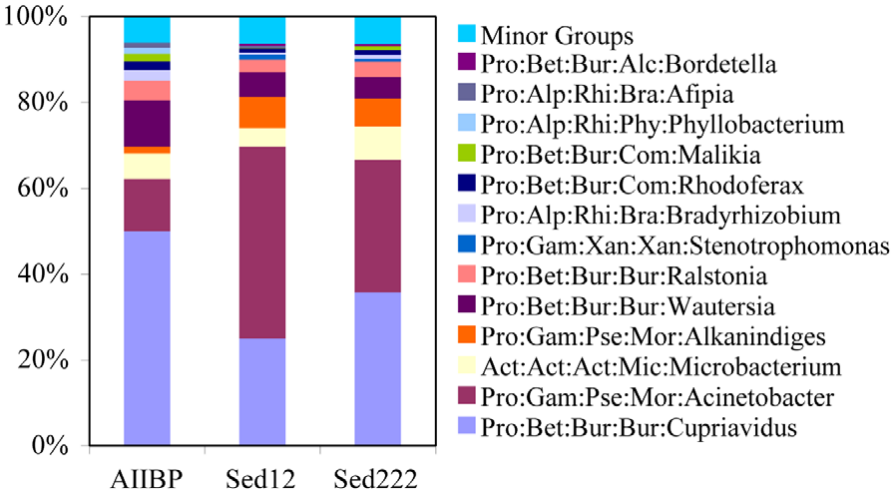
Microbial communities in the three samples. The 16S fragments obtained from the reads were classified in the RDP database. The confidence threshold was not used in the classification. The genus with the highest confidence level was therefore assigned to the fragment. The average confidence level for genera is listed in Table S2. Only genera occupying >1% in one or more samples were included in the figure. All the remaining genera were included in minor groups.

The 16S rRNA gene fragments were extracted from 454 pyrosequencing reads for the AIIBP, Sed12 and Sed222, and were used to identify the major microbial species in the samples. The bacterial compositions of the three samples were similar (Fig. 2). One of the dominant genera in AIIBP, *Acinetobacteria*, was enriched in Sed12 and Sed222. On the other hand, the prevalence of metal-resistant bacteria, including *Cupriavidus*, *Wasteria* and *Ralstonia* in AIIBP, was smaller in the sediment layers (Fig. 2). The compositional similarity between the microbial communities indicates that there may be an active exchange of microbes between the brine and the sediment. The adaptive mechanisms and corresponding genomic changes in the two niches were therefore studied using metagenomic data.

The Estimated Genome Size (EGS) of a metagenome is often an indicator of functional complexity [10]. The estimated EGSs were 7.3 Mb, 9.7 Mb and 9.4 Mb for AIIBP, Sed12, and Sed222, respectively. The EGS of AIIBP was within a reasonable range in reference to the known EGS of the dominant species in the communities. For instance, *Cupriavidus* was the most dominant genus in AIIBP and the EGSs of three known *Cupriavidus* species, *C. necator*, *C. eutropha* and *C. metallidurans*, vary from 6.75 to 7.41 Mb. In the Sed12 and Sed222 samples, the replacement of the dominant genus of *Cupriavidus* by *Acinetobacter* was expected to reduce their EGS, because the three fully sequenced *Acinetobacter* genomes have sizes of 3.4 to 3.9 Mb [28]. But the fact is that the EGSs of Sed12 and Sed222 were larger than the EGS of AIIBP. Considering the high similarity in the microbial communities in the three samples, we speculated that the increase in size was the result of niche-specific genome expansion in the sediments.

### Genome expansion was coupled with gene duplication

**Figure 3:**
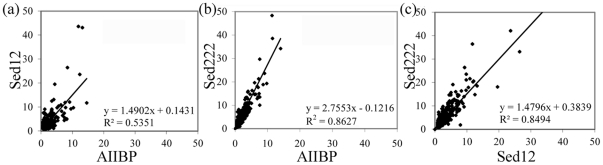
Number of reads for homologs per effective genome. Three sets of read numbers for homologs used for the pairwise comparisons in which AIIBP, Sed12, and Sed222 were involved are shown in Figures 3a–3c, respectively. The homologs have more than 30 reads (longer than 300 bp) in both of the pairwise metagenomes. R values and equations of the correlations are also shown.

Since a bacterial or archaeal genome is almost fully occupied by genes and their derivatives, a genomic expansion is always coupled with duplicated and horizontally transferred genes [29]. To confirm the expected gene duplications in the sediment samples, we calculated the read number for the homologs per effective genome (EG) between all the pairwise matched samples (Fig. 3). The pairwise comparison between AIIBP, Sed12 and Sed222 showed a gradual increase in the read number per EG from AIIBP to Sed222. With respect to the equation in Fig. 3b, Sed222 had about 2.7-fold higher read numbers per EG than AIIBP for these homologs. The read numbers for these samples were highly correlated (see the R values in Figs. 3b–3c), in accordance with high similarity of their communities. Note that the R value in the comparison between Sed12 and AIIBP was lower than that in the two that involved Sed222. These results indicated that gene duplication is genome-wide in these groups of microbes that have penetrated into deeper layers of the sediment, and argues against the duplications being focused on specific sub-sets of genes.

The persistence and decay of the duplicated genes in the metagenomes were examined using z-tests to compare the lengths of the longest putative protein coding sequences (CDSs) identified in the metareads of individual genes. Our results showed that many genes differed significantly in size from the longest CDSs in the metareads (*P*<0.05). The number of cases that had significantly longer CDSs that were found in the reads for homologs in AIIBP was significantly greater than that in Sed12 and Sed222 (chi-square test; P<0.001) (Table S1). Thus, the metagenome of AIIBP was characterized as a group of stable functional genes.

**Figure 4:**
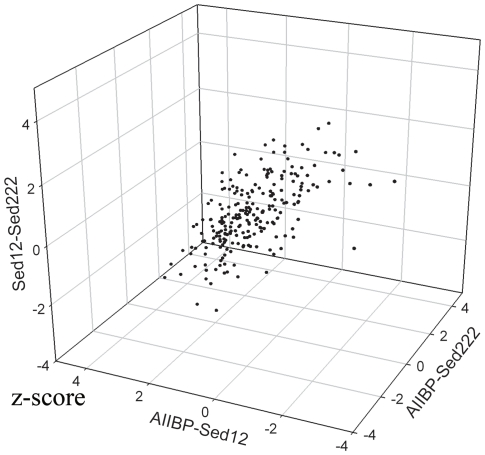
Scores of z-tests using length of the longest ORFs in reads for homologs. A z-test was performed to compare the lengths of the longest unbroken ORFs belonging to the homologs with >30 reads in both metagenomes. The z-scores for the homologs shared by three pairwise comparisons, AIIBP-Sed222, AIIBP-Sed12 and Sed12-Sed222, were plotted.

The common homologs from the AIIBP samples were selected for 3D-plotting of their z-scores in the pairwise comparisons (Fig. 4). Obviously, the points for the z-scores were skewed to greater than 0 for AIIBP-Sed12 and AIIBP-Sed222, on average to 1.4 and 1.53, respectively. This suggests that there were more complete homologs in AIIBP than in the sediment samples. The points for Sed12-Sed222 were mostly around 0, on average −0.55, meaning that the homologs of Sed12 were generally shorter than those of Sed222. All these results suggest that the duplicated genes included many disrupted ones in the sediment samples.

### Highly frequent gene disruptions in the sediment samples

**Figure 5:**
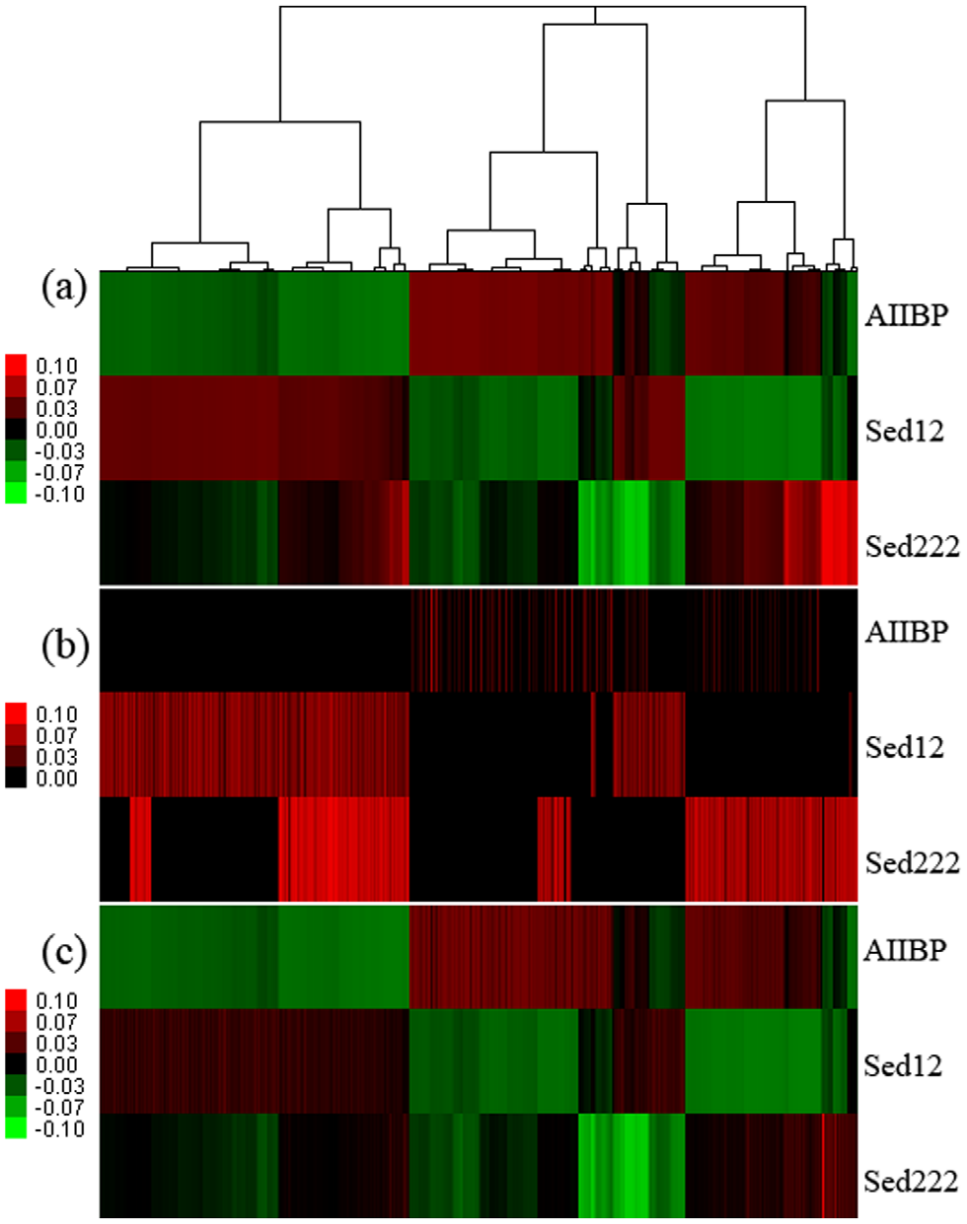
Decay and persistence of abundant genes. A total of 701 homologs (>0.05% of total reads in at least one sample) were used to show their abundance in three samples, AIIBP, Sed12 and Sed222. The genes were clustered by the Cluster 3 program using the complete linkage method after normalization of their abundances (a). The corresponding disruption rates of the homologs are shown in (b). The disrupted genes in the red part of (a) were removed according to the rates in (b). The heatmap (a) was thus modified to (c) showing the adjusted abundance of the homologs after the reduction of the disrupted genes.

To show the prevalence of disrupted genes in the expanded genomes, 701 genes that were highly abundant in separate samples were selected for estimation of the disruption rate. These genes were among those that had undergone heavy duplications in response to some specific environmental changes. The abundance of 701 genes is shown in Fig. 5a, and their disruption rates spanned a wide range of 0–96% (Fig. 5b). The genes in Sed12 and Sed222 were found to be associated with a high disruption rate (Table 1). On average, 47.6% of the abundant genes in Sed12 were predicted as being disrupted, as were 70% of the genes in Sed222.

**Table 1:** Summary of the disruption rates of the abundant genes.

Sample	Gene number	Read length (bp)	ORF length (aa)	RRP (±SD)	Disruption rate (±SD)
AIIBP	339	426	128	1.00 (±0.06)	8.5% (±12.6%)
Sed12	361	268	72	0.85 (±0.07)	47.6% (±11.4%)
Sed222	330	422	95	0.75 (±0.07)	70% (±14.9%)

The genes of high abundance (normalized percentage >0 in Fig. 5a) were used for the estimation of disruption rate. The gene number differed among the samples. The lengths of the reads and the ORFs identified therein were averaged. The RRP is the ratio of real average ORF length to predicted ORF length (see Materials and Methods). The disruption rate was estimated on the basis of a simulation in reference to the RRP value. SD, standard deviation.

In contrast, the abundant genes from AIIBP basically remained intact. According to our simulation-based prediction, 8.5% of the AIIBP genes were disrupted. The heatmap in Fig. 5a shows the abundance of the homologous genes regardless of their functional status. After the fraction of the disrupted genes was removed, the patterns in Fig. 5a changed markedly in Sed12 and Sed222 but not AIIBP (Fig. 5c). The high disruption rates of the genes from the sediments suggested that a considerable fraction of the duplicated genes were dysfunctional pseudogenes or functional variants.

The possible sources of the high disruptions also include pyrosequencing errors. To evaluate the influence of the sequencing errors, the RRP (ratio of the predicted average length of the ORF to the length of the longest undisrupted ORF; see Materials and Methods) was generated using a house-keeping gene that encoded a translational elongation factor (EF-Tu) in the three metagenomes. If the pyrosequencing is accurate, the RRP is greater than 1. Our results showed that AIIBP, Sed12 and Sed222 had high RRPs of 1.08, 0.96 and 1.06, respectively. Both of the rates for the living samples of AIIBP and Sed222 were larger than 1, indicating that the rate of sequencing errors was very low.

### Point mutation-induced gene disruptions

The above estimations of the disruptions were based on simulations and could be affected by the number of the reads and the full length of the identified proteins. To validate the high disruption rates in the sediment samples, we tested various scenarios of disruptions and employed statistics that can infer the causal factors. With the Blast results, we could compare the CDSs in the reads with those in known genes to find the longest matching parts and CDS frameshifts induced by indels. We first studied the genes with a normalized percentage that was greater than 0 in Fig. 5a, which was the same set for the simulation-based disruption measurement. The metareads containing ‘complete’ CDSs occupied 61.6% of the AIIBP KEGG genes, 46% of the Sed12 KEGG genes, and 50.1% of the Sed222 KEGG genes (Table 2). The reads for AIIBP therefore consisted of more complete CDSs than did those for the sediment samples. Moreover, the percentages of CDS ‘shift’ and ‘transverse’ events for Sed12 and Sed222 were also higher than those for AIIBP (Table 2). However, such small percentage differences could not explain the high disruption rates for the sediment samples and the extremely low rates for AIIBP as shown in Table 1. We note that the Blast program ignored the stop codons that can induce CDS disruptions when sequences were aligned with the known proteins. Thus, there could be some premature terminations caused by point mutations in the complete CDSs identified from the metareads of the sediment samples. We next compared the length of the longest CDSs and that of the best Blast matches. The ratio (ROB1) for the combined ‘complete’ and ‘disrupted’ scenarios was close to 1 for AIIBP, but was 0.78 and 0.88 for Sed12 and Sed222, respectively (Table 2), confirming that there were more premature terminations in the sediment samples than in AIIBP.

**Table 2:** Statistics of the disruption styles.

Sample	Complete	Disrupted	ROB1	Transverse	Shift	Unknown	ROB2
Low abundance							
AIIBP	54.9%	24.6%	0.91	0.7%	14%	3.7%	0.22
Sed12	51%	26%	0.87	0.5%	16.9%	5.5%	0.30
Sed222	56.1%	22%	0.87	1.6%	17%	3.3%	0.26
High abundance							
AIIBP	61.6%	23.1%	0.96	0.6%	11.9%	2.7%	0.20
Sed12	46%	26.3%	0.78	1.6%	18.9%	7%	0.33
Sed222	50.1%	28.2%	0.88	1.4%	16.1%	3.9%	0.26

The genes of high abundance are described in Table 1, and those of low abundance are referred to as homologs with a normalized percentage <0, as shown in Fig. 5a. The ORFs and Blast results of the reads for these genes are summarized. The average length of the metareads and the longest ORFs identified within them were first measured for each gene and then used to obtain the average for the 701 genes. On the basis of the Blast results, five scenarios of the matches to known genes were defined: complete, disrupted, transverse, shift, and unknown. ROB1 and ROB2 for a further evaluation of the size of the ORFs were measured (see Materials and Methods).

Sed12 not only had a lower ROB1 than Sed222, it also had lower percentages of ‘complete’ and ‘disrupted’ genes, implying a higher disruption rate in Sed12 than in Sed222. This observation appears to be inconsistent with the simulation-based results, which suggested that the Sed222 genes were more frequently disrupted than those of Sed12 (Table 1). This was perhaps due to more point mutations in ‘disrupted’ and even ‘complete’ genes in Sed12, which might have resulted in shorter CDSs in the reads than expected in addition to difficulties in applying the simulation results to Sed12.

If multiple CDSs belonging to the same gene were identified in a read, the CDSs were generally found to be in a scenario of either ‘transverse’ or ‘shifted’. The ratio (ROB2) for these two scenarios and the others was also calculated. Interestingly, the ratios for Sed12 and Sed222 were higher than those for AIIBP, although the maximum was still very low (only 0.33; Table 2). The higher ratio in Sed12 and Sed222 allows us to infer that the CDSs could extend further for the genes disrupted in Sed12 and Sed222 than for those disrupted in AIIBP, and that these genes might then develop new functions with similar purposes. However, the low ROB2 of all the three samples suggests that the genes in these scenarios were largely pseudogenes. The sites where the shifts of CDSs occurred were found to have 3–6 polynucleotides, suggesting that the shifts were not caused by pyrosequencing errors [30] and that the polynucleotides were hotspots for structural shifts and reorganization of genes. The high disruption rates of the abundant genes in the sediment samples were therefore confirmed by our scenario analysis.

The same analysis was used on genes of low abundance in the samples (normalized percentages less than 0 in Fig. 5a) to determine the disruption rates of genes with small copy numbers. Compared with genes of high abundance, the higher percentage of ‘complete’ (56.1%) and the lower percentage of ‘disruption’ (22%) for these low-abundance genes from Sed222 suggested that the genes with a low copy number were more likely to be functional than the highly duplicated genes in Sed222 (Table 2). Similar results were obtained for Sed12 as well, with 51% ‘complete’ and a ROB1 of 0.87. These results suggest that a high rate of disruptions mainly occurred in the genes of high abundance in the two sediment layers. A reverse trend was observed in AIIBP, which was characterized by a decreased percentage of ‘complete’ and ROB1 genes and an increased percentage of ‘disrupted’ genes.

## Discussion

In this study, we demonstrate genome expansion and gene duplications in natural bacterial communities colonizing the sediments underlying the lower layer of the Atlantis II brine pool - a closed ecosystem. We developed methods to measure the level of gene duplication and to evaluate the gene disruption rate by using metagenomic pyrosequencing data. We then demonstrated niche-specific gene duplications in Sed12 and Sed222, supporting the notion that gene duplication is a fundamental strategy for microbes during their colonization in a new location. Furthermore, we confirmed that gene duplication was a principle source of genome expansion in the depths of the Red Sea.

### Driving forces of gene duplications

For Sed12 and Sed222, we studied the highly disrupted, abundant genes to examine their functions and origins. These genes in Sed222 (>50% disrupted) were mostly integrated into the KEGG pathways of two-component systems, ABC transporters, nitrogen metabolism, homologous recombination, aromatic degradation, and metabolism of nucleotides and amino acids. For the genes in these pathways, AIIBP homologs had significantly fewer reads than Sed222 and Sed12, of which Sed12 had significantly fewer reads than Sed222 (t-test; P<0.0001; Fig. S1). This supported the hypothesis that the genome expansion could be attributed to the duplication of the homologs during their dispersal into the sediment. The high disruption rates also implied that they were still not stable in the microbial genomes.

Although pseudogenes represent one type of duplicated gene and occupy a considerable part of a genome [29], [31], the high fraction of disrupted genes in Sed12 and Sed222 is still beyond expectation. We speculate that this was a result of adaptation to the sediment environments. The total metal contents accounted for 36% of the dry weight of the sediment samples, much higher than that of AIIBP. Heavy metals, including Pb, Cu, Hg, Zn, and Ag, are all toxic to microbial cells [32], [33]. Therefore, it is reasonable to assume that duplication of existing genes encoding ABC transporters to control the uptake and removal of heavy metals was driven by the environment when they migrated from the water column to the sediment. These genes could have been modified to create many structural variants as indicated by high disruption rates, and the transporters produced by functional variants may have been more efficient at pumping out the heavy metal ions. This process is coincident to the typical adaptive radiation model for creation of new gene functions [34].

The temperature difference between the three samples remains unknown, but a previous report suggested that the surface layer of the sediment is somewhat hotter than the overlying brine [35]. Several aromatic compounds were detected in the brine water and were hypothesized to be products resulting from hydrothermal conditions in our previous study [25]. The highest concentration of aromatic compounds might be located in a surface sediment layer like Sed12 where organic debris had accumulated and the temperature was even higher than in AIIBP [35]. The aromatic compounds produced can be used by bacteria such as *Acinetobacteria* and *Alkanindiges*, which explains their higher compositions in Sed12 than in AIIBP (Fig. 2). Therefore, the differences in microbial composition between sediment and water samples might be caused by availability of nutrients and physical/chemical conditions. The presence of aromatic compounds matched the high abundance of the related genes for aromatic degradation in AIIBP and Sed12 [25]. In the deep sediment layer (Sed222), the organic debris was probably largely consumed by hydrothermal reactions and microbes. Correspondingly, the duplicated KEGG genes involved in the aromatic degradation pathways of ko00362, ko00623, and ko00930 were also highly disrupted and pseudogenized. Probably, the absence of some metabolic pathways in Sed222 resulted from the decreased concentration of relevant natural compounds that were efficiently produced in the brine pool and the upper sediment layer.

The related methane monooxygenase gene [36], which encodes the enzyme that catalyzes the first step of the process, was about 29-fold and 19-fold more abundant in Sed12 and Sed222, respectively, than in AIIBP, but was also highly disrupted. The strong methane oxidization process in the sediment was believed to be activated by reduction reactions that use the abundant iron oxides in the sediments according to previous reports [33], [37]. The metal oxides were produced by strong mineralization processes undergoing in AIIBP [38]. Benthic accumulation of the metal oxides possibly induced duplication of the genes responsible for methane oxidization at the surface layer of the sediment.

The duplication of some of the adaptive genes might have occurred in microbial lineages that originate from AIIBP during colonization in the sediment. The accumulation of mutations on these genes would lead to reduction in their expression dosage or complete removal of the highly duplicated genes in response to changes in environmental stress [3], [14]. Indeed, some of these genes might have developed into new genes as previously hypothesized [34]. Different from the soil samples on land surface, our sediment samples contained a small range of microbial lineages and thus horizontal gene transfer between different species cannot provide sufficient gene sources to cope with environmental stresses. This drives the microbes to duplicate existing genes to develop new functions.

Maintaining a balance between persistence, decay and evolution of the duplicated genes is a basic scheme for the fitness of microbes and this fundamentally influences the structural organization of a genome [39]. Decay of copies occurs when microbes colonize into different but stable environmental settings, leading to shrinkages of their genomes. The final outcome is the situation typically found in many genomes where the genome is organized such that half of its genes have single copies, a quarter have two copies, an eighth have three copies, etc. [40]. However, this was not true in our sediment samples, in which the microbes maintained a high proportion of duplicated genes, including pseudogenes and variants with similar or even novel functions. How the microbes maintain and manage such a large genome and the adaptive significance of this process remain unclear.

### Maintenance of a huge genome

In previous studies, large bacterial genomes were characterized as free-living with few pseudogenes and slow generation time [3], [41]. We found that the microbes in Sed12 and Sed222 had large genomes that included many incomplete gene copies. It appeared that the microbes had undergone strong genomic reconstruction and had not yet produced enough generations to modify the redundant elements in the genome. Small genomes can promote reproductive efficiency and thus facilitate the expansion of the population [42]. In ocean microbes, this is perhaps true because there are many eukaryotic predators and competition for natural resources is strong. The sediment in AIIBP is a closed ecosystem in that only the microbes living in the AIIBP had opportunities to colonize there. Low nutrient supply and the absence of predators allow the microbes in the sediment to support large genomes, because they do not need to maintain a small genome to achieve competitive advantages. Instead, we suggest that the amplified genes and their variants in a large genome help these microbes to survive in the new setting.

Because we believe that the genome expansion observed in the AIIBP sediments resulted from gene duplication, we assume that the microbes in Sed222 removed the pseudogenes by recombination in a relatively stable environment. However, this process may have been slow because nutrients derived from organic debris would be expected to be largely consumed by the microbes and hydrothermal reactions in the surface layer [43] and were probably scarce in the deep layers of the sediment. This may result in slow growth of the microbes, uptake and transformation of external DNA and then a slow generation time like that described for soil microbes [3], [41]. A determining step for genome deterioration is genome replication, during which defective genes and redundant fragments are removed by homologous recombination mediated by repeats and transposases [41]. However, with the slow generation time, the redundancy could not be deleted within a short period. Therefore, the microbial genomes in Sed12 and Sed222 remained dynamic and complex. In Sed12, the repertoire of the genes adjusted according to variant conditions, as well as their genomes. Thus, the genomes are likely changing all the time. In comparison, we assume that the genomes of the microbes in Sed222 will gradually become simple and stable under laboratory conditions.

### Decay of DNA in the Sed12 sample

Because Sed12 was close to the surface of the sediment core, there were some dead microbes in the sample. Some of the metareads of Sed12 probably originated from short and highly mutated DNA fragments. This could partially explain a shorter average length of the homologs identified in the metaread of Sed12 (Table 1), compared to those from AIIBP and Sed222. However, the proportion of dead bacteria might be low in the subsurface of the hydrothermal sediment and DNA decay in the DNA sample of Sed12 could not affect the measurement of its EGS. We calculated the EGS for Sed12 and Sed222 again by using the reads longer than 300 bp only, and found that the EGS of Sed12 was still larger than that of Sed222. The result infers that more gene duplications occurred in Sed12 than in Sed222, which is contrary to the finding shown in Fig. 3. It is possible that some groups of genes were specifically duplicated in Sed12 and were not shown in Fig. 3.

To some extent, the gene disruption in Sed12 might also be a result of DNA decay or of point mutations on the metareads derived from dead microbes in this layer of the sediment, as indicated by its unexpectedly low ROB1. In Sed12, the mutations of cytosine to uracil accumulate rapidly under hyperthermal conditions without an active repair system [44], [45]. Evidence of the occurrence of the mutations was found in the lower average G+C content of 50% in the metareads of Sed12, compared to 62% and 57% in those of AIIBP and Sed222, respectively. This result again suggests that most of the microbes in Sed222 were alive and that the relatively high disruption rates of Sed222 genes were not an artifact derived from spontaneous mutations in dead microbes.

In metagenomic analysis, whole genome amplification (WGA) has been shown to raise biased composition of microbial communities and result in inaccurate counting of individual gene copies [46]. In this study, the accuracy of EGS could be affected to some extent. However, a report showed that the composition of principle microbial groups, as well as the repertoire of their genes, was not affected by this amplification treatment [47], suggesting that although WGA treatment could biasedly duplicate some genes, a remarkable overall amplification of the multiple genes in a pathway was unlikely. We used 35 COG genes for the EGS estimation, and identified about 3000 reads for them. Accidental over-amplification of some of these genes had a limited impact on our conclusion. In this study, we used environmental samples to exhibit the microbial niche-specific expansion. Although there was only a short list of dominant genera in the samples, we had yet to determine the degree of the genome expansion that occurred in the individual genera. Culture confirmation and genome sequencing of the strains in question could help to reveal the events that occurred in the bacterial genomes in the new niche.

## Materials and Methods

### Collection of brine water samples

Brine water samples were collected from the Atlantis II Brine Pool (AIIBP; 21°20.76′ N, 38°04.68′ E) during the R/V Oceanus cruise in October 2008 [48]. All necessary permits were obtained for the described field studies. The depth of the sampling sites was greater than 2100 m. At the sampling location, 40 L of brine water were collected from a hydrocast equipped with four 10 L-Niskin bottles connected in series. Temperature and other environmental parameters such as pressure and salinity were recorded by a CTD unit. The samples were immediately filtered through a 1.6 µm GF/A membrane (diameter 125 mm; Whatman, Clifton, NJ, USA) to remove suspended particles and diatoms, and then through a 0.22 µm polycarbonate membrane (diameter 45 mm; Millipore, Bedford, MA, USA) to capture microbial cells. The 0.22 µm polycarbonate membrane was then frozen at −80°C in 0.8 mL of extraction buffer (40 mM EDTA, 0.75 M sucrose, 0.5 M NaCl, 50 mM Tris; pH-8). Total genomic DNA was extracted and purified according to the SDS-based method described elsewhere [49].

A 2.45-meter sediment core was taken from the bottom of the AIIBP during the same cruise. The core was frozen at −80°C before being sliced aseptically into sections of 3 cm. A total of 10 g of sediment from the slices of 12–15 cm (Sed12) and 222–225 cm (Sed222) were used for DNA extraction and purification by using a MO BIO soil DNA isolation kit (Solana Beach, CA, USA).

### 454 pyrosequencing of metagenomes

Because the amount of the isolated crude DNA was not sufficient for 454 pyrosequencing, we had to use a REPLI-g WGA kit (Qiagen, Hilden, Germany) to amplify the microbial genomic DNA from AIIBP and the sediment layers. Amplified DNA was subjected to pyrosequencing using the ROCHE 454 FLX Titanium platform. A total of 991,323, 876,569, and 1,099,605 raw reads with an average length of ∼400 bp were obtained for AIIBP, Sed12 and Sed222, respectively. The 16S rRNA gene fragments in the reads were identified using the rRNA_HMM program [50]. The classification of the fragments into genus level was made in the Ribosomal Database Program (RDP) database [51]. The average confidence level of the classification is shown in Table S2.

### Identification of COG and KEGG genes

The KEGG protein database (v51) (http://www.genome.jp/kegg) encompassing protein sequences from 930 archaeal and bacterial strains was downloaded. Pyrosequencing reads were used for the Blast against the protein databases, with a parameter of “–e 1E-4”. The Blast results were summarized and the best hits were selected as the KEGG genes in the reads. About 0.02% of the reads contained hypothetical eukaryotic genes in Sed12 and Sed222, and the eukaryotic noise was ignored in the subsequent analyses. Percentages of the KEGG genes from the three samples were calculated and 701 genes with at least 0.05% difference in percentage among the samples were selected for clustering in the Cluster 3 program [52]. The percentages were normalized in each sample and then centralized for each gene by relevant mean. The genes of high abundance were thus associated with a positive percentage. The clustering of these genes was conducted by the Cluster 3 program using the complete linkage method. The heat map of the genes was shown by TreeView associated with the Cluster 3 program.

The EGS of a microbe is defined as the combined size of its genome, viral insertions and all plasmids. EGS was calculated by using of the following equation:
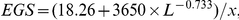
where L is average read length and x is the number of 35 Clusters of Orthologous Groups (COGs) per Mb [10]. The sequences of the 35 COGs were obtained from the STRING database (v. 6.2) and were used to construct a Blast query database. The parameter x is the number of reads with a minimal score of 60 after being Blasted against the query database. The number of reads for a homolog in different samples could be normalized as that in the effective genome (EG) using the following equation:
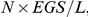
where N is the number of reads and L is the total length of the pyrosequencing reads in megabase.

### Z-test on homologous genes

The numbers of reads for the homologous genes obtained from the three samples were counted. When there were more than 30 reads >300 bp for the homologs, the reads were collected. The length of the longest putative protein coding sequence (CDS) in each read was obtained. The average length of the reads for a homolog was divided by 1000 to adjust the value in 1 kb read. A z-test was performed to compare the revised lengths of the longest CDSs found in the reads from pairwise matched samples.

### Disruption rate of the abundant genes

The reads for the 701 genes used by the Cluster3 program were isolated for further tests on the completeness of the genes. Because the primary focus was on the disruption rate of highly abundant genes, only those with positive relative abundance after the normalization by the Cluster3 were selected. To examine the completeness of these genes in the metagenomes, we performed a simulation by collecting corresponding full-length proteins encoded by these highly abundant genes from the KEGG database and by obtaining the average length of the proteins belonging to 651 different species. The proteins from the dominant archaea and bacteria were considered first; the dominance of the communities was determined by the RDP classification results of the 16S rRNA fragments extracted from the metareads. The protein lengths were then weighted according to their compositions in the microbial communities (Table S3). If a protein was not found in the genomes of all the dominant species, the average length of the homologous proteins from the KEGG database was used. We then constructed three datasets containing the reads of the abundant homologs from the three samples. If all the reads were fully occupied by undisrupted genes, the predicted average length of the ORFs (PO) in the reads was calculated as one-third the average length of these reads. Generally, the real genes in a metagenome are longer than ones in the metareads and thus most of the reads should be located inside genes. But, occasionally, only a part of the read contains the gene, and, in this situation, the PO should be adjusted to minimize the influence of the noncoding regions in the reads. Specifically, considering all the possible overlaps between the reads and the genes, the PO was adjusted using the following formula:
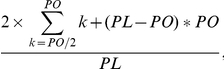
where PL is the length of the protein encoded by the gene. The formula assumed that at least half of a read was occupied by a gene. The adjustment became unnecessary when the PL was less than the PO.

The real average length of the longest undisrupted ORFs (RO) in the reads containing the same genes was then resolved. The best hit in the Blast result was used to determine the correct translation start and strand for the target protein, generating the CDSs. If the ratio of RO to PO (RRP) was less than one, a certain fraction of the gene copies were predicted to be disrupted in the metagenome.

We then estimated the disruption rate of the highly abundant homologous genes in a metagenome. The estimation was based on a simulation that provided a correlation between the gene disruption rate and the RRP ratio. First, a total of 10^5^ artificial genes 3 kb in length were created by adding non-stop random codons and then randomly introduced mutations simultaneously into all of these genes (Fig. S2). The process mimicked the emergence of point mutations in the homologs in a given microbial community during their colonization of a new niche. When the overall mutation rate of these genes reached 0.01%, they were translated into proteins. The disruption rate of these genes and the RRP were recorded. Because there are no overlaps between reads and genes in this simulation, the above PO adjustment was not necessary. The RRP was equal to the average length of the longest CDSs divided by 3000 (the size of the genes). A table containing the disruption rates and their RRPs was generated when the mutational rate was increased by an increment of 0.01%. By finding the RRPs of the genes in the metagenomes in the table, we obtained their approximate disruption rates (Fig. S3).

### Survey of the CDSs in the reads based on Blast results

The CDSs in the reads were further studied to confirm the disruptions predicted by the simulation-based method. We used the best hit of the Blast results against the KEGG database to locate the position of the CDS in known KEGG genes with a threshold score of 60. If a read contained only one CDS, a ‘complete’ scenario for the gene in the read was defined when 1) the CDS occupied 90% of the read; 2) the gene started in the read (the match was initiated from one of the first 10 amino acids (AAs)) and the extent of the match stopped at less than 10% of the read length; and 3) the gene stopped in the read (<10 AAs to the end) and the starting point of the Blast match was within the first 10% of the read. Otherwise, the gene in the read was considered to be ‘disrupted’. When the Blast result of a read contained two or more hits, we defined a ‘transverse’ scenario if the CDSs belonging to the same genes were arranged in head-to-head or tail-to-tail style, or a ‘shift’ scenario if there were insertions or deletions (indels) between the CDSs. The remaining cases in the Blast results were classified as ‘unknown’. Because the CDSs identified by the Blast tool contained stop codons, the difference between the longest undisrupted CDS and that from the Blast result was assessed. The ratio of the length of the longest CDSs to that of the corresponding best Blast matches was calculated for combined scenarios of ‘complete’ and ‘disrupted’ (ROB1); the ratio for the other scenarios was designated as ROB2. The longest CDSs were located on the same translation frame, as indicated by the Blast results.

## Supporting Information

Figure S1 **Average number of the reads for the genes remarkably disrupted in the sediment layers.** The stem-leaf graph was made by using the read numbers of a total of 64 genes in KEGG pathways of ko02020, ko02010, ko00362, ko00280, ko00330, ko03440, ko00623, and ko03420. The number of all the reads for these genes had been normalized to the read number per million reads for a sample. Data are illustrated in stem-leaf plots that contained the median (horizontal line) as well as the 25^th^ and 75^th^ percentiles (bottom and top edges of the boxes). These genes were associated with a high disruption rate of >50% in Sed222.
(TIF)

Figure S2 **Simulation of disruption rates on artificial genes.** Disrupted homologous genes in a metagenome and mutations on their metareads are schematically shown in Fig. S2A. A simulation of gene disruptions is briefly described in Fig. S2B on artificial genes of 1kb. Mutations were randomly generated on the genes and disruption rate was then measured.
(TIF)

Figure S3 **Simulated correlation between RRP and disruption rates using artificial genes.**
(TIF)

Table S1 **Number of pairwise matches of genes having significantly longer ORFs in reads.** Z-tests were performed using the longest ORFs in the metareads for individual orthologous genes with >30 reads. The number of pairwise matched genes is shown for the samples having significantly longer ORFs in the reads (P<0.05).
(DOCX)

Table S2 **Classification confidence of 16S rRNA fragments extracted from metareads.** The confidence values were obtained from the RDP database; the average and standard deviation (SD) are shown in this table. The total number of the 16S fragments in the metagenomes is 792, 620, and 805 for AIIBP, Sed12 and Sed222, respectively.
(DOCX)

Table S3 **Dominant bacteria and their KEGG accession numbers.** The percentages in this table were used to determine the average size of known orthologs in each sample (See Materials and Methods).
(DOCX)
