# Supplementary figures and images for: Bacterial Niche-Specific Genome Expansion Is Coupled with Highly Frequent Gene Disruptions in Deep-Sea Sediments
Source: PLoS One. 2011 Dec 21;6(12):e29149. doi: 10.1371/journal.pone.0029149 (PMC3244439; doi:10.1371/journal.pone.0029149)

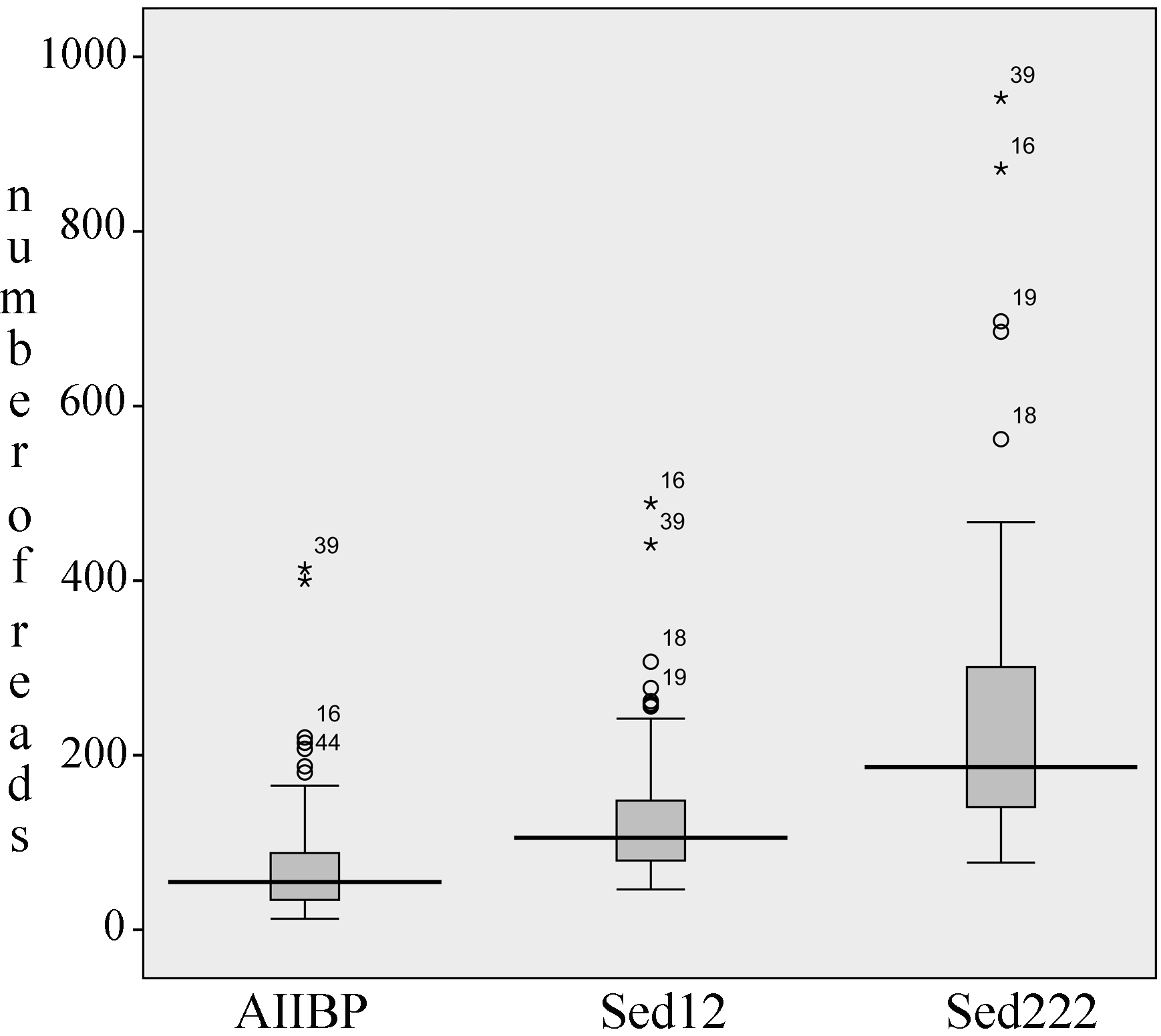

Supplement: Figure S1 — Average number of the reads for the genes remarkably disrupted in the sediment layers. The stem-leaf graph was made by using the read numbers of a total of 64 genes in KEGG pathways of ko02020, ko02010, ko00362, ko00280, ko00330, ko03440, ko00623, and ko03420. The number of all the reads for these genes had been normalized to the read number per million reads for a sample. Data are illustrated in stem-leaf plots that contained the median (horizontal line) as well as the 25th and 75th percentiles (bottom and top edges of the boxes). These genes were associated with a high disruption rate of >50% in Sed222. (TIF) [file pone.0029149.s001.tif]

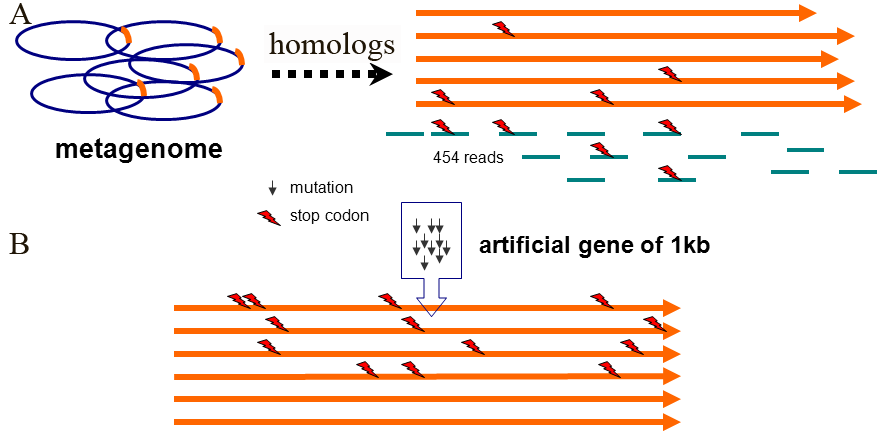

Supplement: Figure S2 — Simulation of disruption rates on artificial genes. Disrupted homologous genes in a metagenome and mutations on their metareads are schematically shown in Fig. S2A. A simulation of gene disruptions is briefly described in Fig. S2B on artificial genes of 1kb. Mutations were randomly generated on the genes and disruption rate was then measured. (TIF) [file pone.0029149.s002.tif]

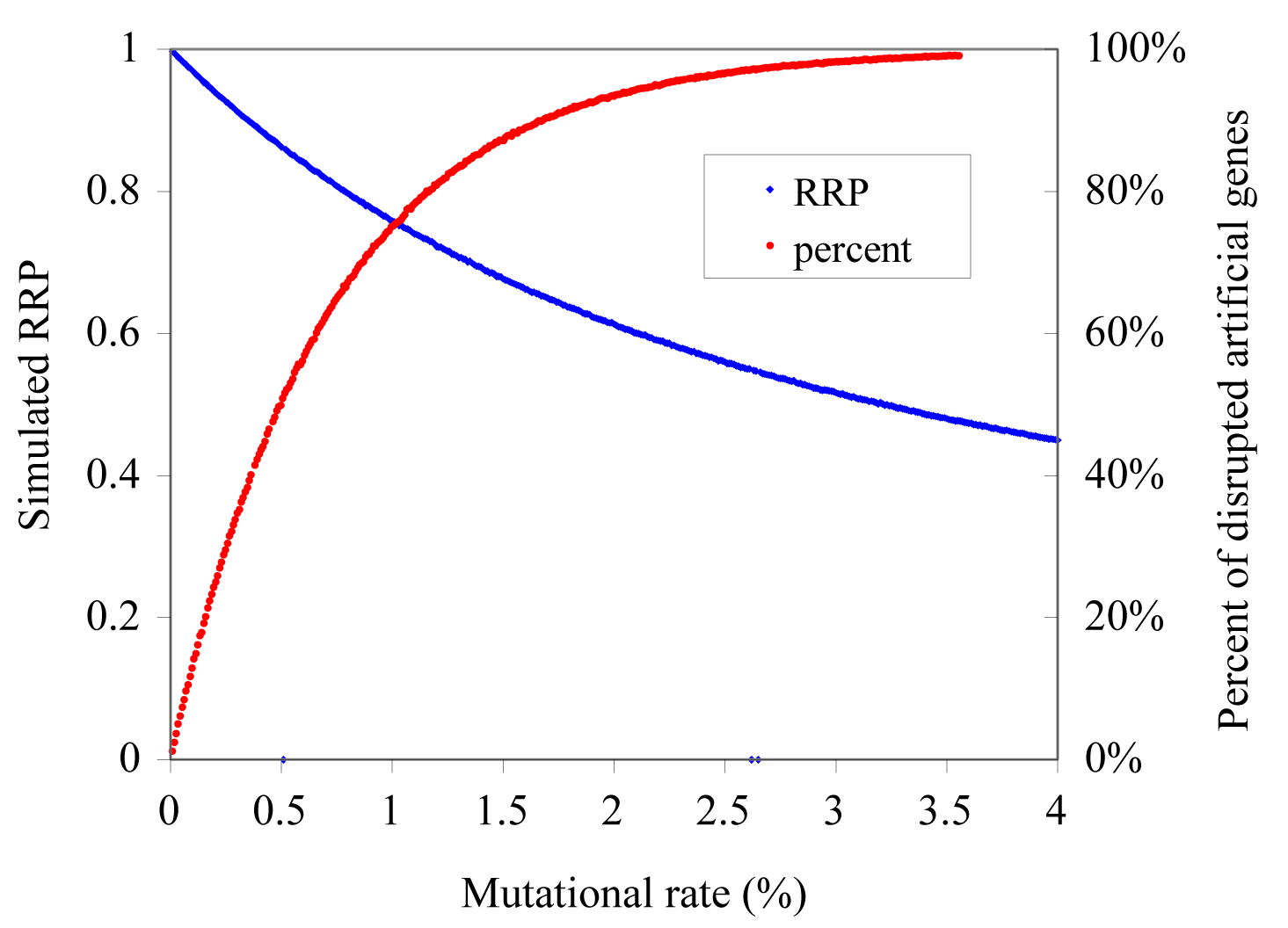

Supplement: Figure S3 — Simulated correlation between RRP and disruption rates using artificial genes. (TIF) [file pone.0029149.s003.tif]
