# Supplementary material for: Bacterial Niche-Specific Genome Expansion Is Coupled with Highly Frequent Gene Disruptions in Deep-Sea Sediments
Source: PLoS One. 2011 Dec 21;6(12):e29149. doi: 10.1371/journal.pone.0029149 (PMC3244439; doi:10.1371/journal.pone.0029149)
Supplement: Table S1 — Number of pairwise matches of genes having significantly longer ORFs in reads. Z-tests were performed using the longest ORFs in the metareads for individual orthologous genes with >30 reads. The number of pairwise matched genes is shown for the samples having significantly longer ORFs in the reads (P<0.05). (DOCX) [file pone.0029149.s004.docx]

Table S1 Number of pairwise matches of genes having significantly longer ORFs in reads

|  | AIIBP | Sed12 | Sed222 |
| --- | --- | --- | --- |
| AIIBP |  | 142 | 129 |
| Sed12 | 9 |  | 10 |
| Sed222 | 3 | 33 |  |

Z-tests were performed using the longest ORFs in the metareads for individual orthologous genes with >30 reads. The number of pairwise matched genes is shown for the samples having significantly longer ORFs in the reads (P<0.05).
