# Supplementary material for: Bacterial Niche-Specific Genome Expansion Is Coupled with Highly Frequent Gene Disruptions in Deep-Sea Sediments
Source: PLoS One. 2011 Dec 21;6(12):e29149. doi: 10.1371/journal.pone.0029149 (PMC3244439; doi:10.1371/journal.pone.0029149)
Supplement: Table S2 — Classification confidence of 16S rRNA fragments extracted from metareads. The confidence values were obtained from the RDP database; the average and standard deviation (SD) are shown in this table. The total number of the 16S fragments in the metagenomes is 792, 620, and 805 for AIIBP, Sed12 and Sed222, respectively. (DOCX) [file pone.0029149.s005.docx]

Table S2 Classification confidence of 16S rRNA fragments extracted from metareads

|  | AIIBP | | Sed12 | | Sed222 | |
| --- | --- | --- | --- | --- | --- | --- |
| Genus | confidence | SD | confidence | SD | confidence | SD |
| Cupriavidus | 90% | 15% | 88% | 17% | 91% | 15% |
| Acinetobacter | 91% | 15% | 90% | 17% | 90% | 17% |
| Microbacterium | 96% | 10% | 93% | 12% | 93% | 16% |
| Alkanindiges | 64% | 13% | 64% | 21% | 65% | 18% |
| Wautersia | 56% | 14% | 58% | 18% | 54% | 18% |
| Ralstonia | 91% | 19% | 83% | 26% | 91% | 22% |
| Stenotrophomonas | - | - | 73% | 31% | 97% | 4% |
| Bradyrhizobium | 93% | 14% | 70% | 49% | 93% | 10% |
| Rhodoferax | 45% | 22% | 67% | 36% | 55% | 29% |
| Malikia | 9% | 2% | - | - | 7% | 3% |
| Phyllobacterium | 97% | 4% | - | - | - | - |
| Afipia | 66% | 19% | - | - | - | - |
| Bordetella | - | - | 99% | 1% | 98% | 3% |
| Meiothermus | - | - | - | - | - | - |
| Methanothermobacter | - | - | - | - | - | - |
| Methanotorris | - | - | - | - | - | - |

The confidence values were obtained from the RDP database; the average and standard deviation (SD) are shown in this table. The total number of the 16S fragments in the metagenomes is 792, 620, and 805 for AIIBP, Sed12 and Sed222, respectively.
