# Supplementary material for: Bacterial Niche-Specific Genome Expansion Is Coupled with Highly Frequent Gene Disruptions in Deep-Sea Sediments
Source: PLoS One. 2011 Dec 21;6(12):e29149. doi: 10.1371/journal.pone.0029149 (PMC3244439; doi:10.1371/journal.pone.0029149)
Supplement: Table S3 — Dominant bacteria and their KEGG accession numbers. The percentages in this table were used to determine the average size of known orthologs in each sample (See Materials and Methods). (DOCX) [file pone.0029149.s006.docx]

Table S3 Dominant bacteria and their KEGG accession numbers

| Bacteria | Accession | AIIBP | Sed12 | Sed222 |
| --- | --- | --- | --- | --- |
| Acinetobacter sp. ADP1 | T00185 | 20% | 65% | 55% |
| Cupriavidus taiwanensis | T00702 | 50% | 25% | 35% |
| Ralstonia metallidurans | T00351 | 30% | 10% | 10% |

The percentages in this table were used to determine the average size of known orthologs in each sample (See Materials and Methods).
